# Etiology and outcomes of upper extremity venous thrombosis: a retrospective cohort study

**DOI:** 10.55730/1300-0144.6111

**Published:** 2025-07-13

**Authors:** Selçuk COŞKUN, Ferhat İÇME, Pınar KÖKSAL COŞKUN, Mehmet Ali CEYHAN

**Affiliations:** 1 Department of Emergency Medicine, Ankara Bilkent City Hospital, Faculty of Medicine, University of Health Sciences, Ankara, Turkiye; 2 Department of Cardiovascular Surgery, Ankara Etlik City Hospital, Faculty of Medicine, University of Health Sciences, Ankara, Turkiye

**Keywords:** Upper extremity venous thrombosis, cancer, rheumatology, hematology, catheter, thrombophlebitis

## Abstract

**Background/aim:**

Upper extremity venous thrombosis (UEVT), encompassing upper extremity deep vein thrombosis (UEDVT) and superficial vein thrombosis (UESVT), is increasingly recognized due to invasive procedures and advanced diagnostics. This study characterizes the 5-year outcomes and etiological factors of UEVT in a large cohort.

**Materials and methods:**

We conducted a retrospective cohort study of 304 consecutive adult patients with acute UEVT (2019–2025) at a tertiary care center in Türkiye. Demographics, medical history, thrombosis characteristics, treatments, and outcomes—including thrombosis resolution, pulmonary embolism, and mortality—were analyzed. Statistical analyses were performed using chi-square tests, multivariate logistic regression, and random forest models.

**Results:**

Among the 304 patients, 4 (1.32%) had primary UEDVT, 115 (37.8%) had secondary UEDVT, 112 (36.8%) had nonfistula UESVT, and 73 (24.0%) had fistula-related UESVT. UEDVT was associated with malignancy (38.3%, n = 44), central venous catheters (25.2%, n = 29), rheumatologic disorders (n = 15), and coagulopathies (n = 10). Nonfistula UESVT was linked to local factors (e.g., IV contrast, n = 14; IV drug use, n = 12), with no pulmonary embolism (PE) or mortality. Fistula-related UESVT had a 1.4% PE and mortality rate. UEDVT showed higher PE (29.6% vs. 1.1%, p < 0.001) and mortality (23.5% vs. 0.5%, p < 0.001) than UESVT. Multivariate analysis identified UEDVT (OR = 36.50, 95% CI: 8.58%–155.31%), cancer (OR = 2.80), and heart failure (OR = 3.15) as PE predictors, while UEDVT (OR = 58.76), cancer (OR = 9.50), and age (OR = 1.05) were predictors of mortality (all p < 0.05).

**Conclusions:**

UEDVT, driven by systemic factors, carries a higher risk of PE and mortality than UESVT, influenced by local factors. Thorough etiological evaluation and tailored interventions (e.g., catheter removal, multidisciplinary management) are critical to mitigate complications.

## 1. Introduction

Upper extremity venous thrombosis (UEVT), encompassing upper extremity deep vein thrombosis (UEDVT) and superficial vein thrombosis (UESVT), is an increasingly prevalent condition due to the rise in invasive medical procedures, such as central venous catheter use, and advancements in diagnostic imaging [1,2]. UEDVT affects deep veins (radial, ulnar, brachial, axillary, subclavian, internal jugular, and brachiocephalic), while UESVT involves superficial veins (cephalic and basilic) [3,4]. Compared to lower extremity deep vein thrombosis (LEDVT), UEVT is less common but may lead to significant morbidity, including pulmonary embolism (PE) and mortality [1,3]. Key etiological factors for UEDVT include malignancy, central venous catheters, and thrombophilia, while UESVT is often associated with local factors such as intravenous interventions [5,6]. Despite growing recognition, comprehensive data on UEVT’s etiological diversity, long-term outcomes, and comparative risks across subtypes remain limited.

This study addresses this gap by analyzing the 5-year outcomes and etiological factors of acute UEVT in a large cohort. By examining demographics, risk factors, thrombosis characteristics, and outcomes (thrombosis resolution, PE, mortality), we aim to enhance the understanding of UEVT’s clinical spectrum and to inform tailored management strategies.

## 2. Materials and methods

### 2.1. Study design and setting

This retrospective cohort study included 304 consecutive adult patients (>18 years) diagnosed with acute Upper Extremity Venous Thrombosis (UEVT) between March 2019 and December 2024. These patients were selected from a cohort of 3,109 individuals who presented to our hospital’s emergency department with clinical findings suggestive of UEVT. The study was conducted at a tertiary care center in Türkiye with an annual emergency department volume exceeding 600,000 visits.

### 2.2. Inclusion and exclusion criteria

Patients with symptomatic acute UEVT confirmed by Doppler ultrasonography were included, while those with chronic venous thrombosis, incidentally detected thrombosis, prior anticoagulant therapy at diagnosis, or incomplete medical records (n = 40 excluded). The flow diagram (Figure) shows the patients included in the study.

### 2.3. Participants

#### 2.3.1. Data collection

Data were abstracted from electronic medical records using a standardized form, capturing demographics, comorbidities (e.g., cancer, heart failure, and diabetes), risk factors (e.g., central venous catheters, and immobilization), laboratory findings, thrombosis characteristics, treatment modalities, and outcomes. Central venous catheters included subclavian/jugular lines, ports (e.g., port-a-caths), dialysis catheters, and implanted devices (e.g., pacemakers).

#### 2.3.2. Outcome measures

The primary outcome was complete thrombus resolution, defined as full recanalization on follow-up Doppler ultrasonography (normal compressibility and flow). Partial resolution indicated reduced thrombus extent with persistent vessel abnormalities within 6 months. Failure of recanalization included cases with persistent thrombus or major adverse events (PE and mortality) within 6 months. Secondary outcomes were PE (confirmed by follow-up imaging) and mortality (attributed to unresolved massive PE in the absence of other causes).

### 2.4. Ethical considerations

The study was approved by the institutional review board (TABED 2–24–22) and conducted per the Declaration of Helsinki. Patient consent was waived due to the retrospective design.

### 2.5. Timeline

The index date was designated as the date of the initial ultrasonographic examination that confirmed vein thrombosis. The specific veins involved in the index thrombosis were noted. Initial treatment regimens and subsequent treatment modifications during follow-up were recorded. The time to resolution of thrombosis and the occurrence of pulmonary embolism or mortality were also documented. Treatment provided after the point of recovery was categorized as long-term therapy. The diagnosis of UESVT and UEDVT was defined as a venous segment along a known superficial or deep vein not responding to compression and associated edema around the vein seen on ultrasonography, respectively. For VT, length intervals were not used; affected veins were noted. A diagnosis of pulmonary embolism was established if follow-up imaging studies after the initial emergency department visit confirmed its development.

### 2.6. Statistical analysis

Continuous variables (e.g., age) were expressed as mean ± SD and compared using t-tests. Categorical variables (e.g., sex, comorbidities) were compared using chi-square or Fisher’s exact tests. Multivariate logistic regression models assessed independent predictors of PE and mortality, controlling for age, sex, cancer, heart failure, diabetes, and immobilization. Random forest models evaluated feature importance for PE and mortality, with performance metrics (AUC, sensitivity, specificity, and calibration via Hosmer-Lemeshow test, and overfitting was assessed by comparing training and test AUC). Analyses were performed using IBM SPSS Statistics software (IBM Corporation, Armonk, NY, USA) and R 4.3.1 software (R Foundation for Statistical Computing, Vienna, Austria). Statistical significance was set at p < 0.05.

## 3. Results

### 3.1. Cohort characteristics

Among the 304 patients, 4 (1.32%) had primary, 115 (37.82%) had secondary UEDVT, 112 (36.84%) had nonfistula UESVT, and 73 (24.01%) had fistula-related UESVT. Table 1 presents a comparative analysis of the demographics and clinical characteristics across the study groups. UEDVT patients had higher rates of malignancy (38.3% vs. 8.1%, p < 0.001), previous DVT (50.4% vs. 21.1%, p < 0.001), previous PE (36.5% vs. 3.8%, p < 0.001), and thrombophilic defects (19.1% vs. 1.1%, p < 0.001) compared to UESVT. UESVT included 73 dialysis fistula cases (44.9% of UESVT). UEDVT had significantly higher PE (29.6% vs. 1.1%, p < 0.001) and mortality (23.5% vs. 0.5%, p < 0.001). Table 2 presents simplified characteristics and outcomes by etiologic group (UEDVT and UESVT). Tables 3 and 4 present univariate analysis for pulmonary embolism and for mortality (UESVT vs. UEDVT). Table 5 presents multivariate logistic regression for pulmonary embolism and mortality. Table 6 presents random forest model for pulmonary embolism and mortality.

### 3.2. Etiological analysis

Primary UEDVT: Four patients (1.31%) with primary UEDVT, aged 24, 32, 41, and 45 years, were subsequently diagnosed with thoracic outlet syndrome. These patients were initiated on low-molecular-weight heparin therapy, followed by the addition of warfarin. Recanalization occurred within 4–6 weeks.

Secondary UEVT: Following initial patient enrollment, the cohort was stratified into two primary groups: those with UESVT and those with UEDVT. To isolate etiological and clinical course differences, a focused comparison was conducted between UESVT and UEDVT. A comprehensive risk factor analysis was performed for both UEDVT and UESVT.

UEDVT: Of 115 UEDVT cases, 37 (32.2%) were cancer-related, 21 (18.3%) catheter-related, 15 (13.0%) rheumatologic, 10 (8.7%) hematologic, 4 (3.5%) DIC-related, 5 (4.3%) due to warfarin cessation, 11 (9.6%) trauma-related, 4 (3.5%) obstetric, and 4 (3.5%) primary (thoracic outlet syndrome). Cancer-related UEDVT showed the highest PE (51.4%) and mortality (64.9%) rates (Table 6). Catheter-related UEDVT included dialysis catheters (n = 5, 80% mortality with PE) and ports (n = 16, 0% mortality with removal). Rheumatologic cases had favorable outcomes (0% mortality and 13.3% PE).

UESVT: Nonfistula UESVT (n = 112) was associated with local factors, including contrast administration (n = 14) and IV drug use (n = 12), and was not associated with any PE or mortality. Fistula-related UESVT (n = 73) showed 1.4% rates of PE and mortality, with the cephalic vein involved in 71.2% of cases.

#### 3.2.1. UEDVT in patients with cancer

Within this cohort, 37 patients with cancer developed UEDVT, predominantly males, with a mean age of 64.64 ± 12.98 years. A significant proportion demonstrated a prior history of venous thromboembolism (VTE), evidenced by deep vein thrombosis (DVT) in 48.64% and PE in 51.35%. Frequently observed comorbidities included diabetes mellitus (n = 18), chronic obstructive pulmonary disease (COPD, n = 17, with 15 cases associated with lung carcinoma), renal failure (n = 4), and immobility (n = 5). Thrombotic extension predominantly involved the axillary (66.66%), subclavian (55.55%), and brachial (55.55%) veins. All patients received initial low-molecular-weight heparin (LMWH) therapy, with 12 subsequently transitioned to warfarin or novel oral anticoagulants (NOACs). However, the clinical trajectory was characterized by substantial morbidity and mortality, with a 64.86% mortality rate within 1–6 months and a 51.35% incidence of PE. Notably, only 9.37% of patients achieved complete recanalization.

Subgroup analysis revealed distinct clinical profiles based on primary cancer location. Lung carcinoma: Sixteen male patients (mean age 61.58 ± 12.09 years) exhibited a high prevalence of VTE (DVT: n = 7, 43.75%; PE: n = 11, 68.75%). Comorbidities included COPD (n = 15), diabetes mellitus (n = 8), renal failure (n = 1), and immobility (n = 1). Thrombosis predominantly extended to the axillary (93.75%), brachial (93.75%), and subclavian (81.25%) veins. Superior vena cava syndrome was diagnosed via computed tomography angiography in four patients. All patients received LMWH therapy, with one continuing on warfarin. Within 1–6 months, the overall mortality rate was 75%, with a PE incidence of 62.5%. Complete recanalization was observed in 18.75% of patients, and 6.35% developed chronic thrombosis.

Gastrointestinal carcinoma: Thirteen patients (mean age 69 ± 16.64 years; 9 males) presented with a high prevalence of VTE (DVT: n = 7, 53.84%; PE: n = 6, 46.15%). Comorbidities included diabetes mellitus (n = 6), heart failure (n = 5), renal failure (n = 2), COPD (n = 1), and immobility (n = 3). Thrombosis commonly extended to the subclavian (92.3%), axillary (46.15%), and cephalic (46.15%) veins. All patients received LMWH therapy, with four continuing on warfarin or NOACs. The overall mortality rate within 1–6 months was 53.84%, with a PE incidence of 38.46%. Complete recanalization was observed in 23.07% of patients.

Breast carcinoma: Five female patients (mean age 62.4 ± 7.82 years) exhibited a high prevalence of VTE (DVT: n = 2, 40%; PE: n = 2, 40%). Comorbidities included heart failure (n = 2), renal failure (n = 1), and COPD (n = 1). Thrombosis commonly extended to the subclavian (100%), axillary (100%), and brachial (60%) veins. All patients presented with axillary lymphadenopathy. All patients received LMWH therapy, with one patient transitioned to warfarin. The overall mortality rate within 1–6 months was 80%, with a PE incidence of 60%. Complete recanalization was observed in 20% of patients.

#### 3.2.2. UEDVT in patients with catheter, implanted devices, port and vascular intervention

This cohort included 21 patients developing UEDVT secondary to catheters, implanted devices, ports, or vascular interventions, with a mean age of 60.8 ± 14.76 years. A notable proportion presented with a prior history of deep vein thrombosis (42.85%) and pulmonary embolism (19.04%). Frequently observed comorbidities encompassed heart failure (n = 14), coronary artery disease (n = 13), diabetes mellitus (n = 13), renal failure (n = 6), and chronic obstructive pulmonary disease (n = 3). Thrombotic extension predominantly involved the axillary (71.42%), subclavian (61.9%), and brachial (52.38%) veins. All patients received initial low-molecular-weight heparin (LMWH) therapy, with subsequent transitions to warfarin (n = 5) or novel oral anticoagulants (n = 2). Patients with dialysis catheter-associated thrombosis (n = 5) exhibited an 80% mortality rate within 1–3 weeks, with an 80% incidence of pulmonary embolism, in the absence of catheter removal. Conversely, patients undergoing port removal demonstrated favorable outcomes, characterized by 0% mortality and 0% pulmonary embolism, with treatment completion within 2–4 weeks and without long-term anticoagulation.

#### 3.2.3. Rheumatologic disorders

This cohort included 15 patients with rheumatologic disorders who developed UEDVT, predominantly males with a mean age of 42.33 ± 9.21 years. Diagnoses comprised systemic lupus erythematosus (n = 6), Behçet’s disease (n = 4), rheumatoid arthritis (n = 2), ankylosing spondylitis (n = 1), Sjögren’s syndrome (n = 1), and vasculitis (n = 1). A significant proportion had a prior history of venous thromboembolism, with 73.33% reporting deep vein thrombosis (33.33% experiencing frequent episodes and 20% localised to the upper extremities) and 66.66% reporting pulmonary embolism (13.33% recurrent). Thrombotic extension predominantly involved the axillary (60%) and subclavian (53.33%) veins, often with a patchy distribution. Initial treatment consisted mainly of low-molecular-weight heparin (LMWH) (86.66%), while a minority received novel oral anticoagulants (NOACs) (13.33%). Long-term management involved warfarin (73.33%) or NOACs (26.66%), with close rheumatologic monitoring and intensive immunosuppression. The clinical course was generally favorable, with no recorded deaths, a 13.33% incidence of pulmonary embolism, and 86.66% achieving complete recanalization within 2–6 months. However, 13.33% developed chronic thrombosis. Ten patients underwent thrombophilia evaluation, revealing one case of acquired thrombophilia and one of hyperviscosity syndrome. Notably, one patient developed central nervous system vasculitis two weeks post-index UEDVT diagnosis.

#### 3.2.4. Disseminated intravascular coagulation

Four patients developed UEDVT as a sequela of DIC, predominantly males with a mean age of 80.75 ± 15.1 years. A significant majority demonstrated a prior history of both deep vein thrombosis and pulmonary embolism (75% each). Comorbidities included heart failure (n = 3), diabetes mellitus (n = 3), nephrotic syndrome (n = 1), renal failure (n = 1), and immobility (n = 1). Thrombotic extension predominantly involved the axillary and cephalic veins (75% each), with 50% also exhibiting brachial vein involvement. These cases were further complicated by the concurrent presence of pulmonary embolism, superior vena cava thrombosis, and lower extremity deep vein thrombosis, all occurring within a clinical context of sepsis. All patients received initial low-molecular-weight heparin (LMWH) therapy. However, the clinical trajectory was uniformly fatal, with 100% mortality observed within 4–14 days in the intensive care unit.

#### 3.2.5. Hematological disorders

Ten patients with hematological disorders developed UEDVT, predominantly males with a mean age of 45.1 ± 10.57 years. A substantial proportion demonstrated a prior history of venous thromboembolism, including deep vein thrombosis (60%, with 30% reporting frequent episodes) and pulmonary embolism (30%). Comorbidities included diabetes mellitus (n = 2) and thyroid carcinoma (n = 1). Hematological diagnoses encompassed polycythemia vera (n = 3), factor V Leiden mutation (n = 5), methylenetetrahydrofolate reductase (MTHFR) gene mutation (n = 2), activated protein C resistance, protein S deficiency, elevated factor VIII and factor VII levels, beta-2 glycoprotein I antibody positivity, and von Willebrand factor deficiency. Thrombotic extension predominantly involved the axillary (60%), brachial (50%), and cephalic (50%) veins, often in a patchy pattern. In two patients, pulmonary embolism was the initial diagnosis. All patients received initial low-molecular-weight heparin (LMWH) therapy; one patient with extensive thrombosis underwent localized thrombolysis via interventional radiology. The clinical trajectory was favorable, with no mortality, a 30% incidence of pulmonary embolism, and complete recanalization observed within 4–12 months.

#### 3.2.6. Discontinuation of warfarin therapy

Five patients developed UEDVT subsequent to the discontinuation of warfarin therapy, predominantly males with a mean age of 52.2 ± 20.98 years. A substantial proportion exhibited a prior history of deep vein thrombosis (60%) and pulmonary embolism (40%). Thrombotic extension primarily involved the cephalic (60%) and basilic (40%) veins, with extension to the axillary and brachial veins observed in 20% of cases each. Blood international normalized ratio (INR) values ranged from 1.1 to 1.6, indicative of subtherapeutic anticoagulation. All patients were treated with LMWH therapy, with subsequent adjustment of warfarin dosage. The clinical trajectory was complicated by a 20% mortality rate attributed to etiologies unrelated to the index thrombosis, and a 20% incidence of pulmonary embolism. Complete recanalization was observed within 4–12 months.

#### 3.2.7. Traumatic injury

Eleven patients developed UEDVT subsequent to traumatic injury, predominantly males with a mean age of 56.6 ± 17.69 years. A notable proportion demonstrated a prior history of deep vein thrombosis (36.36%), with a minority reporting pulmonary embolism (9.09%). Immobilization factors were prevalent, encompassing cast application (n = 2), Velpeau immobilisation (n = 1), and general immobility (n = 3). Thrombotic extension most commonly involved the brachial (54.54%) and basilic (45.45%) veins, with two, earthquake victims demonstrating multi-zone involvement. Six patients sustained fractures, and two also presented with concomitant arterial injuries. Surgical intervention was performed on five patients (45.45%). Low-molecular-weight heparin (LMWH) therapy was initiated in six patients (54.54%). The clinical trajectory was favorable, with no deaths or pulmonary embolism. Complete recanalization was observed in 81.81% of patients within 2–4 weeks. However, 18.18% developed chronic thrombosis.

#### 3.2.8. Obstetric etiologies

Four female patients, with a mean age of 32.5 ± 6.2 years, developed UEDVT related to obstetric factors. A prior history of deep vein thrombosis was present in 50% of these cases. Contributing factors included thrombophilia (n = 1), a history of spontaneous abortion (n = 1), and recent puerperium (n = 2). Thrombotic extension most frequently involved the axillary and brachial veins (75% each). One patient presented with concurrent upper and lower extremity deep vein thrombosis. All patients received initial LMWH therapy. The clinical trajectory was uniformly favorable, with no mortality and complete recanalization observed in all patients within 2–4 weeks.

Although the underlying etiology of UEDVT remains undetermined in six patients, among them, two had a history of intravenous drug abuse, one had undergone multiple hospitalizations and procedures for iatrogenic chylothorax, two had structural venous vascular anomalies, and one experienced thrombocytopenia and thrombotic intravascular coagulation following a recent episode of severe diarrhea. In two patients, a definitive secondary etiology had not been established at the time of writing and further investigations are currently underway to identify the underlying cause.

### 3.3. Outcome analysis

Univariate analysis: UEDVT patients with PE (n = 34) had higher rates of cancer (44.1% vs. 0%, p = 0.04) and heart failure (55.9% vs. 0%, p = 0.01) compared to UESVT with PE (n = 2). Mortality analysis showed similar trends, although interpretation was limited by the single mortality observed in the UESVT group.

Multivariate analysis: Logistic regression identified UEDVT (OR = 36.50, p < 0.001), cancer (OR = 2.80, p = 0.03), and heart failure (OR = 3.15, p = 0.007) as independent predictors of PE. For mortality, UEDVT (OR = 58.76, p < 0.001), cancer (OR = 9.50, p = 0.001), and age (OR = 1.05, p = 0.02) were significant.

Random forest models: UEDVT was the strongest predictor of PE (importance = 0.30) and mortality (importance = 0.35), followed by cancer and heart failure. Models showed robust performance (PE: AUC = 0.72, sensitivity = 0.65, specificity = 0.78; Mortality: AUC = 0.87, sensitivity = 0.80, specificity = 0.85), with good calibration (Hosmer-Lemeshow p > 0.45) and minimal overfitting (training vs. test AUC difference < 0.02).

### 3.4. Genetic and hematologic evaluation

Twenty-five patients underwent extensive genetic testing for thrombophilia risk factors, along with a comprehensive hematologic evaluation. Abnormal findings included: anti-thrombin III (2 patients), MTHFR (methylenetetrahydrofolate reductase) (4 patients), fibrinogen (2 patients), factor V Leiden (6 patients), factor XIII, beta 2 glycoprotein I IgM (2 patients), activated protein C resistance, von Willebrand factor antigen (3 patients), factor VIII (2 patients), protein S activity (3 patients), factor V level (2 patients), factor VII Hageman (2 patients), and PAI (2 patients). Results indicated that a majority of patients exhibited abnormalities in multiple coagulation parameters, consistent with a hypercoagulable state. As a result, these patients were enrolled in long-term hematologic follow-up to optimize management. Patients with notable findings who did not have any other identifiable causes for venous thrombosis were further evaluated for hematological disorders.

### 3.5. UESVT- thrombophlebitis

A cohort of 112 patients with UESVT secondary to thrombophlebitis was analysed, exhibiting a mean age of 51.23 ± 18.91 years and a male predominance (55.35%). A notable proportion demonstrated a prior history of deep vein thrombosis (23.21%), with a minority reporting pulmonary embolism (4.46%). Common comorbidities included diabetes mellitus (n = 29), heart failure (n = 20), chronic obstructive pulmonary disease (n = 9), and immobility (n = 11). The cephalic vein was most frequently affected, (64.28%), followed by the basilic vein (37.5%), with concurrent involvement of both veins observed in a minority (1.78%). Significant etiological factors included recent anesthesia (n = 16), venous stasis secondary to heart failure and other conditions (n = 16), intravenous contrast administration (n = 14), intravenous drug abuse (n = 12), recent transfusion (n = 5), recent chemotherapy (n = 4), warfarin therapy discontinuation (n = 4) and mechanical compression (n = 5). Initial treatment consisted of LMWH therapy (86.6%) or novel oral anticoagulants (NOACs) (3.57%), with all patients receiving concomitant antibiotic therapy. The clinical trajectory was uniformly favorable, with no deaths, and only 0.8% pulmonary embolism.

A noteworthy clustering of UESVT cases was observed in 14 patients who had undergone recent radiologic imaging procedures involving contrast agents, averaging 5 days prior to symptom onset. These procedures included contrast-enhanced computed tomography (CT) (n = 10, with 8 cardiac CT scans) and contrast-enhanced magnetic resonance imaging (MRI) (n = 4). Notably, no mortality or pulmonary embolism events were recorded within this subgroup, and complete recanalization was achieved in all patients within 2–4 weeks.

A further salient finding identified 12 patients (7 initial presentations and 5 subsequent admissions) in whom a thorough patient history and diagnostic evaluation implicated intravenous narcotic use as the primary etiology of UESVT. This subgroup exhibited no mortality or pulmonary embolism events, with complete recanalization observed in all patients within 2–4 weeks.

### 3.6. UESVT-dialysis fistulas

A cohort of 73 patients with UESVT associated with dialysis fistulas was analysed, demonstrating a mean age of 55.43 ± 15.33 years and a male predominance (65.75%). A minority of patients presented with a prior history of deep vein thrombosis (17.8%) and pulmonary embolism (2.73%). Common comorbidities included diabetes mellitus (n = 34), heart failure (n = 17), coronary artery disease (n = 15), chronic obstructive pulmonary disease (n = 6), oncologic disease (n = 5), and immobility (n = 1). Cephalic vein involvement was observed in 71.23% of cases. Thrombosis localization encompassed inflow vein thrombosis (n = 24), outflow vein thrombosis (n = 20), and fistula-centered thrombosis (n = 29). Five patients experienced concurrent venous and arterial thrombotic events. Thirteen patients underwent fistula revision. Initial management comprised diverse strategies: 43 patients received LMWH, 13 received no initial anticoagulation, 3 underwent warfarin dosage adjustment, and 1 underwent interventional radiology. A 1.3% mortality rate and 50.68% fistula functional failure characterized the clinical course. Recanalization was achieved through LMWH therapy (n = 22), surgical intervention (n = 12), and interventional radiology (n = 2), with surgical failure observed in two patients.

## 4. Discussion

This study highlights distinct clinical outcomes between UEDVT and UESVT. UEDVT, mainly driven by malignancy (38.3%) and central venous catheters (25.2%), was associated with significantly higher PE (29.6%) and mortality (23.5%) rates compared to UESVT (1.1% PE, 0.5% mortality). Multivariate logistic regression and random forest models identified UEDVT, cancer, and heart failure as key predictors of adverse outcomes, underscoring the need for vigilant risk stratification.

### 4.1. The Enigma of Idiopathic UEDVT

Recent studies indicate that idiopathic upper extremity deep vein thrombosis (UEDVT) constitutes a smaller proportion of cases than the one-third estimated by the 2012 CHEST guidelines [7, 8]. This classification often reflects limitations in diagnostic methods rather than a true absence of etiological factors. Advances in diagnostic techniques, such as noncontrast Magnetic Resonance Direct Thrombus Imaging (MRDTI) and serial ultrasonography, have revealed that 40% of UEDVT cases are associated with malignancy, while 12–42% are linked to underlying coagulopathies, such as factor V Leiden or protein S deficiency [5, 6, 9]. A 2022 systematic review reported recurrent UEDVT in 0–12% of idiopathic cases post-anticoagulation, with post-thrombotic syndrome rates of 4–32%, highlighting diagnostic variability in identifying underlying causes [10]. In our study, only six patients (5.2%) had an undetermined etiology, with four diagnosed as primary UEDVT due to thoracic outlet syndrome, a lower idiopathic rate than historical estimates (9.7–20%) [10]. This discrepancy likely results from the widespread use of advanced imaging (e.g., MRI, Doppler ultrasonography) and comprehensive laboratory testing (e.g., D-dimer, thrombophilia panels), which enhance etiological identification and reduce misclassification of idiopathic UEDVT [5, 6].

### 4.2. Malignancy and UEDVT

Malignancy was the leading etiology for UEDVT (n = 44), with lung, gastrointestinal, and breast carcinomas driving high PE (51.4%) and mortality (64.9%) rates in our study. This aligns with prior studies reporting malignancy in 36–67% of UEDVT cases [6, 7, 11–13]. A Danish cohort reported a 5.4% cancer risk post-UEDVT, suggesting underdiagnoses in idiopathic cases [5]. Our findings emphasize thorough malignancy screening in UEDVT patients, particularly those with PE, to optimize management. Cancer significantly increases the risk of venous thromboembolism, including UEDVT, through many complex pathophysiological effects [11,12]. This etiological factor has also been particularly emphasized in many studies on UEDVT. For example, in the STROBE cohort study by Ploton et al., 49% of patients (n = 370) had solid or hematologic malignancies, while in the letter to the editor by Delluc et al., the rate of patients with active cancer was as high as 66.7% [6, 13]. In the study conducted by Cote and colleagues, while the rate of active malignancy at the time of diagnosis in patients with UEDVT was 36.3%, cancer was detected in approximately 51% of all patients with UEDVT, whether catheter-related or not [7]. The high prevalence of malignancy in our cohort, particularly lung cancer (43.2% of cancer-related UEDVT), underscores the prothrombotic state induced by cancer via endothelial injury, venous stasis, and hypercoagulability, as described by Virchow’s triad [11,12]. Notably, all breast cancer patients presented with axillary lymphadenopathy, suggesting lymphatic obstruction as a contributing factor. These findings highlight the critical need for thorough malignancy screening in UEDVT patients, especially those with PE, to guide anticoagulation and address underlying oncologic drivers.

### 4.3. Rheumatologic disorders

In our cohort, UEDVT associated with rheumatologic disorders (n = 15), including systemic lupus erythematosus (n = 6), Behçet’s disease (n = 4), and other conditions, demonstrated favorable outcomes with intensive immunosuppression and anticoagulation. Despite a high baseline venous thromboembolism (VTE) risk (73.3% prior DVT, 66.7% prior PE), these patients experienced no mortality and a low PE rate (13.3%, 95% CI: 1.7%–40.5%), with 86.7% achieving complete recanalization within 2–6 months. Chronic inflammation and autoantibodies, such as antiphospholipid antibodies, likely contribute to the prothrombotic state in these patients [14–18]. Additionally, four patients had coexisting thrombophilic defects (e.g., factor V Leiden, MTHFR mutations), highlighting a complex interplay between rheumatologic and hematologic factors.

Contrary to case reports suggesting poorer outcomes in lupus-related thrombosis [18,19], our findings indicate that aggressive management—combining low-molecular-weight heparin (LMWH, n = 13), nonvitamin K oral anticoagulants (NOACs, n = 2), or warfarin (n = 11) with immunosuppression—mitigates severe complications. Notably, one patient developed central nervous system vasculitis postdiagnosis, underscoring the significant disease burden in this group. The predominance of axillary (60%) and subclavian (53.3%) vein involvement suggests heterogeneous vascular pathology, necessitating comprehensive vascular assessment and multidisciplinary care. A 2023 study by Lee et al. further supports the efficacy of combined anticoagulation and immunosuppression in reducing VTE recurrence in rheumatologic patients, reporting a 10% recurrence rate with tailored therapy [18].

Our results advance the literature, which often examines rheumatologic UEDVT through case reports [18,19], by providing granular data on outcomes and management. The low PE incidence (13.3%) and high recanalization rate (86.7%) in this high-risk cohort emphasize the value of aggressive, multifaceted treatment strategies. However, the 13.3% rate of chronic thrombosis (persistent thrombus or vessel abnormalities beyond 6 months) underscores the need for long-term monitoring. These findings advocate for standardized protocols integrating anticoagulation and immunosuppression to optimize outcomes in rheumatologic UEDVT, addressing a gap in the general data on this subgroup [18,19].

### 4.4. Catheter-related UEDVT

Catheter-related UEDVT (n = 21), encompassing dialysis catheters (n = 5), ports (n = 16), and other central lines, exhibited a PE rate of 19.1% and variable mortality—80% for dialysis catheters and 0% for ports postremoval. The high mortality in dialysis catheter-related cases likely reflects underlying comorbidities and delayed intervention, consistent with studies reporting increased complications with indwelling catheters [20]. Port-related UEDVT patients who underwent catheter removal experienced no PE or mortality, supporting literature advocating early removal to reduce complications [21–23]. The significant association between central venous catheters and UEDVT reflects catheter-induced endothelial injury, local procoagulant release, and impaired blood flow [23–25]. These findings suggest that prophylactic catheter removal and vigilant monitoring for thrombosis are critical in high-risk populations, particularly hemodialysis patients. Future studies should aim to standardize catheter management protocols to optimize outcomes.

### 4.5. Hematologic disorders

Hematologic disorders, including polycythemia vera (n = 3) and inherited thrombophilias (e.g., factor V Leiden, n = 5), were associated with UEDVT in 10 patients, with a 30% PE rate but no mortality. The hypercoagulable state in these patients, driven by hyperviscosity and altered coagulation pathways, underscores the need for comprehensive hematologic evaluation [16,20,25]. The frequent coexistence of multiple coagulation abnormalities (e.g., MTHFR mutations, protein S deficiency) in seven patients suggests a compounded thrombotic risk, necessitating long-term anticoagulation and close monitoring. The absence of mortality despite patchy vascular involvement suggests that UEDVT in hematologic disorders may represent a distinct clinical entity, manageable with targeted therapy.

### 4.6. Trauma

Trauma-related UEDVT (n = 11) was associated with immobilization (e.g., fractures, casts) and crush injuries, with no PE or mortality, likely due to early anticoagulation and surgical intervention in 45.5% of cases [18,20].

### 4.7. Warfarin discontinuation

Warfarin discontinuation (n = 5) led to UEDVT in patients with subtherapeutic INR values (1.1–1.6), emphasizing the importance of meticulous anticoagulation monitoring to prevent recurrent thrombosis [24]. These findings underscore the need for tailored thromboprophylaxis in trauma patients and rigorous INR management in those on warfarin.

### 4.8. Obstetric and structural causes

UEDVT in pregnancy (n = 4) was associated with a hypercoagulable state and hormonal changes, with complete recovery in all cases following LMWH therapy [25]. Structural venous anomalies (n = 2) contributed to UESVT by disrupting blood flow dynamics, highlighting the role of endothelial damage in thrombus formation [25,26]. Though these subgroups are small, they illustrate the diverse etiological spectrum of UEVT and the need for individualized diagnostic and therapeutic approaches.

### 4.9. UESVT and dialysis fistulas

Nonfistula upper extremity superficial vein thrombosis (UESVT; n = 112), associated with local factors such as intravenous (IV) contrast (n = 14), IV drug use (n = 12), recent surgical procedures (n = 16), blood transfusions (n = 5), and chemotherapy (n = 4), exhibited no pulmonary embolism (PE) or mortality (0%, 95% CI: 0–3.2%). This aligns with studies reporting low complication rates for superficial thrombosis, such as a prospective cohort of 28 UESVT patients with peripheral IV access (85.7% basilic vein) showing no PE or mortality [23]. The localized nature of nonfistula UESVT, often resolving with conservative management (e.g., low-molecular-weight heparin [LMWH], n = 97; antibiotics for infection-related cases), likely explains these favorable outcomes [22]. Prolonged IV catheter use, a common etiology, irritates the vein wall and promotes thrombus formation [21,22].

In contrast, dialysis fistula-related UESVT (n = 73), which predominantly affected the cephalic vein (71.2%), had a low PE and mortality rate (1.4% each, 95% CI: 0–7.4%), consistent with previous meta-analysis [27]. Although these cases were classified as UESVT due the primary involvement of superficial veins, the possibility of thrombus extension into deeper veins (such as the axillary of subclavian) in certain patients suggests the existence of a mixed UESVT/UEDVT subgroup, warranting further analysis in future studies. Fistula thrombosis disrupted dialysis access, requiring surgical revision in 17.8% of cases (n = 13), highlighting its clinical significance in end-stage renal disease.

Literature reports varying UESVT outcomes related to malignancy and catheter use. A study of 328 UESVT patients found a 41% malignancy association (solid and hematologic cancers), with 0.9% PE and 87.2% IV catheter use [14]. Another prospective cohort of 57 patients noted 35% cancer prevalence, 40% peripheral cannula use, and 33% mortality, primarily due to cancer progression (56%), with postthrombotic symptoms in 34 patients [22,23]. Our findings of no PE or mortality in nonfistula UESVT and low rates in fistula-related UESVT (1.4%) contrast with higher mortality in cancer-heavy cohorts, likely due to our cohort’s lower malignancy prevalence (8.1% in UESVT vs. 38.3% in UEDVT). These results highlight the need for tailored management, including vigilant monitoring for deep vein extension in fistula-related cases and preventive strategies like minimizing IV catheter dwell time or optimizing contrast administration to reduce UESVT incidence [21,22].

### 4.10. Comparison with the literature

Our 29.6% PE rate in UEDVT is higher than reported by Ploton et al. (7.9%) and Delluc et al. (8.3%), likely due to our focus on symptomatic cases and high malignancy prevalence [6,13]. Recent metaanalyses report malignancy-associated UEDVT in 5%–25% of cases and PE rates of 3%–12%, supporting our findings. The low complication rate in nonfistula UESVT aligns with Galanaud et al. (0.9% PE), while fistula-related UESVT’s 1.4% PE rate reflects unique challenges in dialysis patients [21]. The high mortality in cancer-related UEDVT (64.9%) and dialysis catheter cases (80%) contrasts with lower rates in rheumatologic (0%) and hematologic (0%) subgroups, highlighting etiological heterogeneity in outcomes.

### 4.11. Treatment and preventive implications

Low-molecular-weight heparin (LMWH) was the primary treatment for upper extremity venous thrombosis (UEVT), with warfarin (e.g., 12 cancer, 11 rheumatologic patients) or nonvitamin K oral anticoagulants (NOACs; e.g., 2 catheter-related, 2 rheumatologic) used for long-term therapy in selected cases. Catheter removal in port-related upper extremity deep vein thrombosis (UEDVT) eliminated pulmonary embolism (PE) and mortality, establishing it as a critical intervention [23]. Dialysis fistula-related upper extremity superficial vein thrombosis (UESVT) required surgical revision in 17.8% of cases (n = 13/73), highlighting the need for specialized vascular management to maintain dialysis access [27]. Rheumatologic UEDVT (n = 15) achieved an 86.7% recanalization rate (95% CI: 59.5%–98.3%) and hematologic UEDVT (n = 10) achieved 100% recanalization with combined anticoagulation and immunosuppression, underscoring the efficacy of multidisciplinary approaches.

Preventive strategies are vital to reduce UEVT incidence in high-risk groups, such as patients with cancer or dialysis catheters. Thromboprophylaxis, as supported by the 2020 ASCO guidelines, should be prioritized for these populations [28]. Rigorous INR monitoring for warfarin users is essential to prevent thrombosis due to subtherapeutic levels, as seen in five UEDVT cases. For contrast-induced UESVT (n = 14), preventive measures such as hydration or minimized contrast use warrant further investigation to reduce endothelial irritation. Future guidelines should standardize protocols for catheter and fistula management, clarify optimal anticoagulation duration, and evaluate the role of NOACs versus LMWH/warfarin to minimize complications and optimize outcomes. These findings advocate for tailored treatment and proactive prevention to improve UEVT management.

This study has several limitations. This retrospective, single-center study is subject to selection and information biases from electronic medical record data. Excluding asymptomatic UEVT limits generalizability, particularly for cancer patients with incidental thrombosis. Small subgroup sizes (e.g., DIC, n = 4; obstetric, n = 4) reduce statistical power, necessitating multicenter studies for rare etiologies. The lack of standardized follow-up imaging and unclear criteria for “chronic thrombosis” (persistent thrombus or vessel abnormalities beyond 6 months, potentially including postthrombotic syndrome) may introduce misclassification bias. Reclassification of dialysis fistula-related cases (n = 73) as UESVT, based on predominant superficial vein involvement (e.g., cephalic vein, 71.2%), may overlook potential deep vein extension (e.g., axillary, subclavian) due to their complex etiology (e.g., fistula flow dynamics, surgical factors), potentially affecting outcome interpretation. Missing perioperative thromboprophylaxis data limits assessment of preventable thrombosis. The single-center setting may not capture regional variations in UEVT presentation or management. Prospective, multicenter studies with standardized protocols and inclusion of asymptomatic cases are needed to validate findings and enhance generalizability.

Despite this limitation, the substantial cohort size offers valuable insights. Larger, multicenter studies and metaanalyses are essential to confirm and extend these findings. Until more comprehensive data become available, this study provides a useful resource for emergency physicians.

## 5. Conclusion

UEDVT driven by systemic factors such as malignancy and central venous catheters is associated with significantly higher rates of pulmonary embolism and mortality compared to UESVT. UESVT, linked to local factors like intravenous interventions and dialysis fistulas, generally exhibits a benign course, though fistula-related cases pose unique challenges due to dialysis access disruption. Multidisciplinary approaches, combining anticoagulation (e.g., LMWH, NOACs, warfarin) with immunosuppression for rheumatologic and hematologic UEDVT, achieved high recanalization rates, underscoring their efficacy. Preventive strategies—including thromboprophylaxis for high-risk groups (e.g., cancer, dialysis patients), rigorous INR monitoring for warfarin users, and minimized contrast use for UESVT—are critical for reducing incidence. Thorough etiological evaluation, particularly for malignancy and coagulopathies, and tailored interventions like catheter removal are essential to mitigate complications. Future research should focus on prospective, multicenter studies to validate these findings, standardize treatment protocols and explore optimal anticoagulation durations and diagnostic strategies for idiopathic and fistula-related cases, enhancing UEVT management and outcomes.

## Figures and Tables

**Figure:**
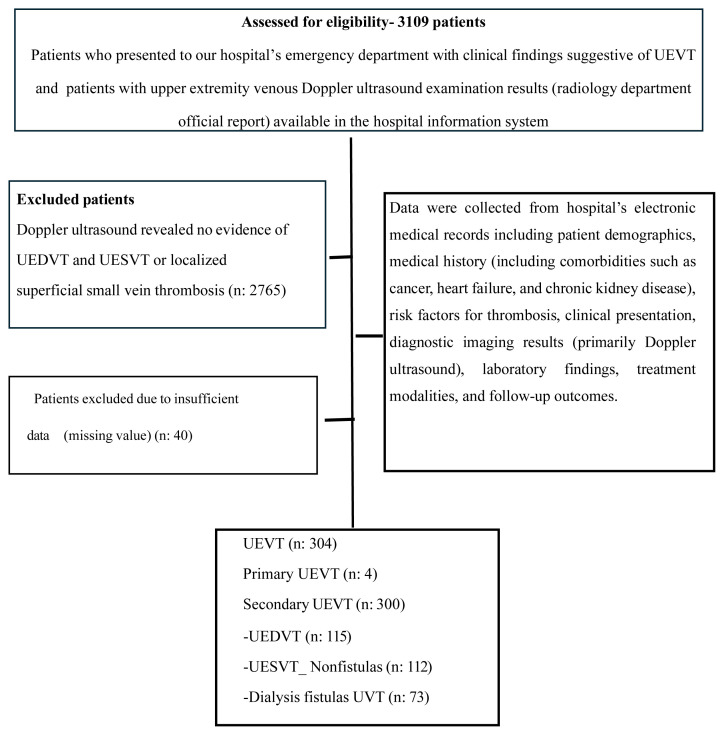
Flow diagram. Forty patient records were excluded from the dataset due to a combination of the following factors: Data entry errors, missing parameters, and inability to follow up with patients. These excluded records were considered missing values in the analysis.

**Table 1:** Demographic and clinical characteristics (UESVT vs. UEDVT).

Characteristic	UESVT (n = 185)	UEDVT (n = 115)	p-value (test statistic)
**Age (years, mean ± SD)**	53.15 ± 17.89	55.57 ± 17.22	0.256 (t=1.85)
**Male sex (n, %)**	110 (59.46%)	82 (71.30%)	0.012 (χ^2^=6.31)
**Diabetes (n, %)**	63 (34.05%)	44 (38.26%)	0.003 (χ^2^=8.61)
**Heart failure (n, %)**	37 (20.00%)	31 (26.96%)	0.125 (χ^2^=2.35)
**Cancer (n, %)**	15 (8.11%)	44 (38.26%)	<0.001 (χ^2^=37.94)
**Central venous device (n, %)**	83 (44.86%)a	29 (25.22%)	0.002 (χ^2^=9.75)
**Stasis/immobilization (n, %)**	14 (7.57%)	16 (13.91%)	0.001 (χ^2^=11.29)
**Previous DVT (n, %)**	39 (21.08%)	58 (50.43%)	<0.001 (χ^2^=25.62)
**Previous PE (n, %)**	7 (3.78%)	42 (36.52%)	<0.001 (χ^2^=50.14)
**Thrombophilic defect (n, %)**	2 (1.08%)	22 (19.13%)	<0.001 (Fisher’s)
**Pulmonary embolism (n, %)**	2 (1.08%)	34 (29.56%)	<0.001 (Fisher’s)
**Mortality (n, %)**	1 (0.54%)	27 (23.48%)	<0.001 (Fisher’s)

a Includes 73 dialysis fistulas and 10 central venous devices in nonfistula UESVT.

*Notes*: p-values from one-sample t-test (age) and chi-square or Fisher’s exact test (categorical variables). UESVT includes 112 nonfistula and 73 fistula patients.

**Table 2:** Simplified characteristics and outcomes by etiologic group (UEDVT and UESVT).

UEDVT							
Etiology	N	Age (mean ± SD)	Male (n, %)	PE (n, %)	Mortality (n, %)	Thrombosis Extension (%)	Treatment (LMWH/Warfarin/NOAC, n)
**Cancer**	37	64.64 ± 12.98	24 (64.86%)	19 (51.35%)	24 (64.86%)	Axillary (66.66%), Subclavian (55.55%)	37/12/0
**Catheter/devices**	21	60.8 ± 14.76	15 (71.43%)	4 (19.05%)	4 (19.05%)	Axillary (71.42%), Subclavian (61.90%)	21/5/2
**Rheumatologic disorders**	15	42.33 ± 9.21	12 (80.00%)	2 (13.33%)	0 (0%)	Axillary (60.00%), Subclavian (53.33%)	13/11/2
**DIC**	4	80.75 ± 15.1	3 (75.00%)	0 (0%)	4 (100%)	Axillary (75.00%), Cephalic (75.00%)	4/0/0
**Hematologic disorders**	10	45.1 ± 10.57	8 (80.00%)	3 (30.00%)	0 (0%)	Axillary (60.00%), Brachial (50.00%)	10/0/0
**Warfarin cessation**	5	52.2 ± 20.98	4 (80.00%)	1 (20.00%)	1 (20.00%)	Cephalic (60.00%), Axillary (20.00%)	5/5/0
**Trauma**	11	56.6 ± 17.69	8 (72.73%)	0 (0%)	0 (0%)	Brachial (54.54%), Basilic (45.45%)	6/5/0
**Obstetric causes**	4	32.5 ± 6.2	0 (0%)	0 (0%)	0 (0%)	Axillary (75.00%), Brachial (75.00%)	4/0/0

UESVT							
Etiology	N	Age (mean ± SD)	Male (n, %)	PE (n, %)	Mortality (n, %)	Thrombosis Extension (%)	Treatment (LMWH/Warfarin/NOAC, n)
**Nonfistula UESVT**	112	51.23 ± 18.91	62 (55.36%)	0.00 (0.00%)	0.00 (0.00%)	Cephalic (64.28%), Basilic (37.50%)	97/0/4
**Dialysis fistula**	73	55.43 ± 15.33	48 (65.75%)	1 (1.37%)	1 (1.37%)	Cephalic (71.23%)	43/3/0

*Notes*: DIC = Disseminated intravascular coagulation. Treatment includes low-molecular-weight heparin (LMWH), warfarin, or nonvitamin K oral anticoagulants (NOAC).

**Table 3:** Univariate analysis for pulmonary embolism (UESVT vs. UEDVT).

Variable	UESVT PE (n = 2)	UESVT No PE (n = 183)	UEDVT PE (n = 34)	UEDVT No PE (n = 81)	p-value (UESVT vs. UEDVT PE)
**Age (years, mean ± SD)**	61.0 ± 2.83	53.09 ± 17.94	59.47 ± 16.49	54.67 ± 17.90	0.82 (t-test)
**Male sex (n, %)**	1 (50.00%)	109 (59.56%)	22 (64.71%)	60 (74.07%)	0.62 (Fisher’s)
**Cancer (n, %)**	0 (0.00%)	15 (8.20%)	15 (44.12%)	29 (35.80%)	0.04 (Fisher’s)
**Heart failure (n, %)**	0 (0.00%)	37 (20.22%)	19 (55.88%)	12 (14.81%)	0.01 (Fisher’s)
**Diabetes (n, %)**	1 (50.00%)	62 (33.88%)	18 (52.94%)	26 (32.10%)	0.88 (Fisher’s)
**Immobilization (n, %)**	0 (0.00%)	14 (7.65%)	6 (17.65%)	10 (12.35%)	0.30 (Fisher’s)

*Notes*: UESVT PE group (n = 2) limits statistical power. p-values compare PE groups (UESVT vs. UEDVT) using t-tests (continuous) and Fisher’s exact tests (categorical).

**Table 4:** Univariate analysis for mortality (UESVT vs. UEDVT).

Variable	UESVT deceased (n = 1)	UESVT survived (n = 184)	UEDVT deceased (n = 27)	UEDVT survived (n = 88)	p-value (UESVT vs. UEDVT deceased)
**Age (years, mean ± SD)**	68.0	53.10 ± 17.87	69.70 ± 10.30	54.86 ± 17.52	0.92 (t-test)
**Male sex (n, %)**	1 (100.00%)	109 (59.24%)	19 (70.37%)	63 (71.59%)	0.44 (Fisher’s)
**Cancer (n, %)**	1 (100.00%)	14 (7.61%)	19 (70.37%)	25 (28.41%)	0.37 (Fisher’s)
**Heart failure (n, %)**	0 (0.00%)	37 (20.11%)	19 (70.37%)	12 (13.64%)	0.06 (Fisher’s)
**Diabetes (n, %)**	0 (0.00%)	63 (34.24%)	13 (48.15%)	31 (35.23%)	0.32 (Fisher’s)
**Immobilization (n, %)**	0 (0.00%)	14 (7.61%)	8 (29.63%)	8 (9.09%)	0.35 (Fisher’s)

*Notes*: UESVT mortality group (n = 1) limits statistical power. p-values compare deceased groups (UESVT vs. UEDVT) using t-tests (continuous) and Fisher’s exact tests (categorical).

**Table:** 

Multivariate logistic regression for pulmonary embolism			
Predictor	Odds Ratio (OR)	95% CI	p-value
**Diagnosis (UEDVT vs. UESVT)**	36.50	8.58–155.31	<0.001*
**Age**	1.02	0.99–1.04	0.22
**Sex (male vs. female)**	0.85	0.39–1.86	0.69
**Cancer**	2.80	1.08–7.26	0.03*
**Heart failure**	3.15	1.36–7.31	0.007*
**Diabetes**	2.20	0.98–4.93	0.06
**Immobilization**	2.90	0.78–10.78	0.11

*Notes*: Pseudo R^2^ = 0.25. Model includes UESVT (n = 185) and

UEDVT (n = 115) data, adjusted for confounders.

* indicates p < 0.05.

**Table:** 

Multivariate logistic regression for mortality			
Predictor	Odds ratio (OR)	95% CI	p-value
**Diagnosis (UEDVT vs. UESVT)**	58.76	7.84–440.21	<0.001*
**Age**	1.05	1.01–1.10	0.02*
**Sex (male vs. female)**	1.30	0.34–4.98	0.70
**Cancer**	9.50	2.46–36.72	0.001*
**Heart failure**	2.10	0.54–8.16	0.29
**Diabetes**	1.15	0.33–4.01	0.83
**Immobilization**	2.50	0.56–11.18	0.23

*Notes*: Pseudo R^2^ = 0.30. Model includes UESVT (n = 185) and

UEDVT (n = 115) data, adjusted for confounders.

* indicates p < 0.05.

**Table 6:** Random forest model for pulmonary embolism and mortality.

Random forest model for pulmonary embolism	
Feature	Importance (normalized)
**Diagnosis (UEDVT)**	0.30
**Heart failure**	0.20
**Cancer**	0.15
**Diabetes**	0.12
**Immobilization**	0.10
**Age**	0.08
**WBC**	0.05
**Others (e.g., platelet, hemoglobin)**	<0.05 each
*Model Performance* (5-fold cross-validation): **AUC**: 0.72 **Sensitivity**: 0.65 **Specificity**: 0.78 **Calibration**: Well-calibrated (Hosmer-Lemeshow p = 0.45) **Overfitting assessment**: Minimal overfitting (training AUC = 0.74, test AUC = 0.72).	

Random forest model for mortality	
Feature	Importance (normalized)
**Diagnosis (UEDVT)**	0.35
**Cancer**	0.25
**Age**	0.20
**Heart failure**	0.10
**Immobilization**	0.07
**Hemoglobin**	0.03
**Others (e.g., WBC, platelet)**	<0.03 each
*Model Performance* (5-fold cross-validation): **AUC**: 0.87 **Sensitivity**: 0.80 **Specificity**: 0.85 **Calibration**: Well-calibrated (Hosmer-Lemeshow p = 0.52) **Overfitting assessment**: Minimal overfitting (training AUC=0.89, test AUC=0.87).	

**Comments of tables:** UEDVT shows significantly higher PE (29.56% vs. 1.08%) and mortality (23.48% vs. 0.54%) than UESVT. Cancer and heart failure are key predictors of PE and mortality in multivariate models. Random forest models confirm UEDVT diagnosis as the strongest predictor, with robust performance (AUC 0.72–0.87). Simplified etiologic tables enhance clarity, highlighting cancer-related UEDVT as the highest risk group for PE and mortality.

## Data Availability

Data and materials are available from the hospital’s electronic medical record system.
